# Gene Expression Analysis of Astrocyte and Microglia Endocannabinoid Signaling during Autoimmune Demyelination

**DOI:** 10.3390/biom10091228

**Published:** 2020-08-24

**Authors:** Álvaro Moreno-García, Ana Bernal-Chico, Teresa Colomer, Alfredo Rodríguez-Antigüedad, Carlos Matute, Susana Mato

**Affiliations:** 1 Department of Neurosciences, University of the Basque Country UPV/EHU, E-48940 Leioa, Spain; alvaro.morenog@ehu.es (Á.M.-G.); ana.bernal@ehu.es (A.B.-C.); alfredo.r.antiguedad.z@gmail.com (A.R.-A.); carlos.matute@ehu.es (C.M.); 2 Achucarro Basque Center for Neuroscience, E-48940 Leioa, Spain; teresa.colomer@achucarro.org; 3 Centro de Investigación Biomédica en Red sobre Enfermedades Neurodegenerativas (CIBERNED), E-28031 Madrid, Spain; 4 Biocruces, Bizkaia, E-48903 Barakaldo, Spain

**Keywords:** multiple sclerosis, EAE, astrocytes, microglia, endocannabinoids

## Abstract

The endocannabinoid system is associated with protective effects in multiple sclerosis (MS) that involve attenuated innate immune cell responses. Astrocytes and microglia are modulated by endocannabinoids and participate in the biosynthesis and metabolism of these compounds. However, the role of neuroglial cells as targets and mediators of endocannabinoid signaling in MS is poorly understood. Here we used a microfluidic RT-qPCR screen to assess changes in the expression of the main endocannabinoid signaling genes in astrocytes and microglia purified from female mice during the time-course of experimental autoimmune encephalomyelitis (EAE). We show that astrocytes and microglia upregulate the expression of genes encoding neurotoxic A1 and pro-inflammatory molecules at the acute disease with many of these transcripts remaining elevated during the recovery phase. Both cell populations exhibited an early onset decrease in the gene expression levels of 2-arachidonoylglycerol (2-AG) hydrolytic enzymes that persisted during EAE progression as well as cell-type-specific changes in the transcript levels for genes encoding cannabinoid receptors and molecules involved in anandamide (AEA) signaling. Our results demonstrate that astrocytes and microglia responses to autoimmune demyelination involve alterations in the expression of multiple endocannabinoid signaling-associated genes and suggest that this system may regulate the induction of neurotoxic and pro-inflammatory transcriptional programs in both cell types during MS.

## 1. Introduction

MS is regarded as a chronic demyelinating disease initiated by pathogenic autoimmune responses against myelin, followed by a broader inflammatory and neurodegenerative process [1]. Local innate immune responses driven by microglia and astrocytes are crucially involved in MS onset and progression [2,3,4]. Classically activated microglial cells produce a plethora of pro-inflammatory molecules that promote leukocyte recruitment and cytotoxicity to oligodendrocytes and neurons. Nevertheless, microglia have also demonstrated unconventional activities with regard to MS, such as increased phagocytosis of myelin debris and secretion of trophic factors that facilitate myelin repair [5,6,7]. Similarly, astrocytes activated during demyelination also acquire complex phenotypes that can both hinder or support central nervous system (CNS) recovery. In the last years, however, it has become clear that these cells play fundamental pathogenic roles during MS. In EAE, reactive astrocytes promote inflammation and neurodegeneration through multiple mechanisms, which include neurotoxicity, the recruitment of inflammatory cells to the CNS, and the modulation of microglial responses [2,4,8]. In this context, recent landmark studies have allowed the characterization of a population of neurotoxic astrocytes expressing complement component 3 (C3) in response to pro-inflammatory cytokines released by activated microglia [9]. This subset of reactive astrocytes, termed A1, exhibit transcriptional programs destructive to synapses and oligodendrocytes and inhibitory to remyelination with potential pathogenic consequences in human MS and animal models of the disease [9,10,11].

The endocannabinoid system is a neuromodulatory system formed by cannabinoid CB_1_ and CB_2_ receptors, their endogenous ligands named *endocannabinoids*, and the enzymes responsible for the production and metabolism of these compounds. The most accepted pathways for the biosynthesis of the two major endocannabinoids AEA and 2-AG involve reactions catalyzed by N-arachidonoylethanolamine phospholipase D (NAPE-PLD) and diacylglycerol lipase α/β isoforms (DGLα/β), respectively [12]. Regarding metabolism, fatty acid amide hydrolase (FAAH) is the principal AEA hydrolase in nervous tissue, although the physiological actions of this and other *N*-acylethanolamides can also be terminated by alternative lipid amidases, such as N-acylethanolamine acid amidase (NAAA) [13]. On the other hand, 2-AG is preferentially metabolized by the serine hydrolases monoacylglycerol lipase (MAGL), α/β-hydrolase domain containing 6 (ABHD6) and 12, (ABHD12) [14]. More recently, a family of fatty acid binding proteins (FABPs) has been identified as an intracellular carrier that delivers endocannabinoids, mostly AEA, to their catabolic enzymes [15]. The synthesis and degradation of endocannabinoids are tightly regulated processes that determine the extent of CB_1_ and CB_2_ mediated signaling. In this context, it is well established that pharmacological or genetic inactivation of AEA and 2-AG hydrolytic enzymes and FABPs augments CNS endocannabinoid levels and engages cannabinoid receptor-dependent (and independent) effects in vivo [13,16,17,18].

The brain endocannabinoid system is centrally involved in the pathogenesis of MS as supported by two main lines of evidence. First and most important, cannabinoid agonists and endocannabinoid hydrolysis inhibitors exert beneficial effects in experimental models of MS that include neuroprotection and attenuated inflammatory responses mediated by adaptative and/or innate immune cells [19,20,21,22,23,24]. Secondly, a number of studies have reported dysregulated levels of endocannabinoids and endocannabinoid signaling elements in MS patients and animal models of the disease. In terms of cannabinoid receptors, both decreased and unaltered CB_1_ receptor levels have been reported in the EAE model [25,26], while increased CB_2_ receptor gene and protein expression is a consistent finding in rodent models of MS [23,24,27]. Results concerning endocannabinoid levels are also often controversial, but the majority of the studies report increased concentrations of AEA [28,29,30,31] and unaltered or enhanced 2-AG levels [21,24,32]. In all, most of these studies support the hypothesis that altered endocannabinoid signaling may modulate MS initiation and progression but do not establish the cell-type specificity of endocannabinoid activity dysregulation in MS. Astrocytes and microglia are endowed with cell-specific dotation’s of CB_1_ and CB_2_ receptors and endocannabinoid synthetic/metabolic enzymes [33,34], but the specific role of these cellular populations as targets and mediators of endocannabinoid signaling in MS remains to be fully characterized.

In this study we investigated changes in the expression of endocannabinoid system genes in astrocytes and microglia associated with their phenotypic transformation during EAE. Our results show convergent but also cell-type specific deregulation of transcripts encoding cannabinoid receptors and endocannabinoid synthetic/metabolic enzymes in both cell populations. Noteworthy, the expression changes affecting endocannabinoid signaling-related genes in astrocytes and microglial cells take place early during EAE and parallels or even precedes the over-expression of genes associated to neurotoxicity and inflammation. These results demonstrate that activated innate immune cells display a deregulated transcriptional profile of endocannabinoid system genes during MS pathology.

## 2. Materials and Methods

### 2.1. Mice

Female C57BL/6 mice for EAE experiments were purchased from Envigo. Animals were maintained under standard laboratory conditions with food and water *ad libitum*. The maintenance of the animal facility and use of animals was in full compliance with the European Communities Council Directive of 22 September 2010 on the protection of animals used for scientific purposes (Directive 2010/63/EU). All animal procedures were approved by the local ethical committee (Comité de Ética en Experimentación Animal, CEEA-UPV/EHU).

### 2.2. Induction of EAE

Myelin oligodendrocyte glycoprotein 30–35 (MOG_30–35_) (MEVGWYRSPFSRVVHLYRNGK), corresponding to the fragment of the mouse MOG from amino acids 35 to 55 was synthesized at the Peptide Synthesis Core Facility of the Pompeu Fabra University. Mice were immunized subcutaneously in the flank with 200 μg MOG_30–35_ peptide in incomplete Freund’s adjuvant supplemented with 8 mg/mL *Mycobacterium tuberculosis* H37Ra (Difco Laboratories). Pertussis toxin (500 ng; Calbiochem, Merck Millipore) was injected intraperitoneally on the day of immunization and again two days later. EAE was induced in a total number of 20 mice that were immunized in four independent experimental sessions separated 2–5 days each (*n* = 5 mice per EAE experiment). Bodyweight and motor symptoms were recorded daily and scored from 0 to 8 as follows: 0, no detectable changes in muscle tone and motor behavior; 1, flaccid tail; 2, paralyzed tail; 3, impairment or loss of muscle tone in hindlimbs; 4, hindlimb hemiparalysis; 5, complete hindlimb paralysis; 6, complete hindlimb paralysis and loss of muscle tone in forelimbs; 7, tetraplegia; and 8, moribund.

### 2.3. Flow Cytometry

On days 7–8, 14–17, and 28–31, naïve and EAE mice were decapitated under isoflurane anesthesia (IsoVet^®^, B Braun, Barcelona, Spain), and cells purified from forebrain and spinal cord tissue according to previously described procedures [21]. Mice were selected for fluorescence-activated cell sorting (FACS) analysis so that each experimental group included animals immunized in 2–3 independent EAE experiments. Briefly, brain tissue was dissected and placed in enzymatic solution (116 mM NaCl, 5.4 mM KCl, 26 mM NaHCO_3_, 1 mM NaH_2_PO_4_, 1.5 mM CaCl_2_, 1 mM MgSO_4_, 0.5 mM EDTA, 25 mM glucose, 1 mM L-cysteine) with papain (3 U/mL) and DNAse I (150 U/μL, Invitrogen) for digestion at 37 ℃ for 25 min. Halfway through the incubation, the minced tissue was triturated 10 times using 5 mL serological pipettes. Following enzymatic digestion, cells were mechanically released by gentle passage through 23 G, 25 G, and 27 G syringe needles. After homogenization, tissue clogs were removed by filtering the cell suspension through prewetted 40 μm cell strainers (Fisherbrand^TM^; Thermo Fisher Scientific, Loughborough, UK) to a 50 mL Falcon tube quenched by 5 mL of 20% heat-inactivated fetal bovine serum in Hank’s Balanced Salt Solution (HBSS, Thermo Fisher Scientific). The cell strainers were thoroughly rinsed with 15 mL HBSS in and cell suspensions were centrifuged 200× *g* for 5 min. To purify astrocytes and microglia from myelin debris, cells were resuspended in 25% isotonic Percoll PLUS (GE Healthcare Europe GmbH, Freiburg, Germany) in HBSS and centrifuged at 200× *g* without brake for 20 min at room temperature. The myelin top layer was aspirated and cells washed with HBSS to remove any traces of Percoll PLUS by centrifuging at 200 × *g* for 5 min. The total dissociated single cells were resuspended in 500 μL sorting buffer (25 mM HEPES, 5 mM EDTA, 1% BSA, in HBSS) containing Normal Rat Serum (1:100; Invitrogen; 10710C) and TruStain FcX™ (anti-mouse CD16/32) antibody (1:100; BioLegend; 101320). The cell suspension was then divided into two equivalent aliquots for the parallel purification of astrocytes and microglia. Isolated CNS cells were incubated with fluorochrome conjugated antibodies ACSA-2-PE (1:50; Miltenyi Biotec, Madrid, Spain; REA969), A2B5-488 (1:100, R&D Systems, Minneapolis, MN, USA; FAB1416G), CD11b-FITC (1:200; BioLegend, San Diego, CA, USA; 101205), CD31-VioBright 515 (1:200; Miltenyi Biotec; REA784), CD45-FITC (1:200; Miltenyi Biotec; REA737) and O1-488 (1:100; R&D Systems; FAB1327G) for astrocyte isolation. Microglia were purified from a cell suspension aliquot incubated with antibodies to CD11b-FITC (1:100; BioLegend; 101205) and CD45-PE (1:100; BioLegend; 103105). In all cases, cell suspensions were incubated with LIVE/DEAD Fixable Green Dead Cell (1:1500; Thermo Fisher).

Astrocytes were sorted as ACSA-2^+^ cells following the exclusion of contaminating microglia, hematopoietic cells, oligodendrocytes, oligodendrocyte progenitors, and endothelial cells grouped in a dump channel [35,36] (Figure S1). Microglia were sorted as CD11b^+^ cells with low CD45 expression [37]. Samples were run on a BD FACS Jazz (2B/4YG) flow cytometer (BD Bioscience) controlled using the BD FACS™ Software (version 1.1.0.84), and the results were analyzed using the FlowJo software. All gatings were set based on appropriate isotype controls. The debris and cell clumps were initially gated out on the basis of forward (FSC) and side (SSC) scatter plots, allowing selection of only the population of interest. Further doublets were gated out using FSC/trigger pulse width plots. The purity of astrocytes was assessed by FACS analysis of sorted ACSA-2^+^ cells following fixation and immunostaining with a C-terminal antibody against the astrocytic marker glutamate aspartate transporter 1 (GLAST) [38] (1:200; kindly donated by JD Rothstein). Astrocytes sorted from naïve and EAE mice were found to be 93–97% positive for GLAST (Figure S1). We further confirmed that we had isolated a relatively pure population of astrocytes and microglia by RT-qPCR analysis for the expression of several cell-type specific markers (Figure S1).

### 2.4. Nanofluidic RT-qPCR in Purified Astrocytes and Microglia

Expression analysis of target genes was carried out by RT-qPCR. An average number of 70,000–90,000 astrocytes and 15,000–20,000 microglial cells per mouse were collected in lysis buffer (Qiagen) containing 1% β-mercaptoethanol for optimal template preservation and stored at −80 ℃ until processing. RNA from the FACS-sorted cells was extracted using the RNeasy plus Micro Kit (Qiagen). The synthesis of cDNA, pre-amplification, and amplification steps were performed at the General Genomics Service of the UPV/EHU following the quality control of RNA samples (2100 Bioanalyzer, Agilent Technologies). For nanofluidic qPCR, the pre-amplified cDNA samples were measured with no reverse transcriptase and no template controls in the BioMark HD Real-Time PCR System using 96.96 and 48.48 Dynamic Arrays of integrated fluidic circuits (Fluidigm Corporation, San Francisco, CA, USA). This technology enables reliable and accurate gene expression analysis using very limited total RNA input [39]. We used commercial primers from the IDT Integrated DNA Technologies or Fluidigm Corporation (Table S1). Data pre-processing and analysis were completed using the Fluidigm Melting Curve Analysis Software 1.1.0 and Real-time PCR Analysis Software 2.1.1 (Fluidigm Corporation) to determine valid PCR reactions. *Gapdh*, *Hprt*, *Ppia,* and *B2m* were included as candidate reference genes for normalization purposes. Data were corrected for differences in input RNA using the geometric mean of the reference genes selected according to the results from geNorm (https://genorm.cmgg.be/) and Normfinder (https://moma.dk/normfinder-software) algorithms. Relative expression values were calculated with the 2^-ΔΔCt^ method.

### 2.5. Immunohistochemistry

For histological examination, anesthetized naïve and EAE mice (*n* = 3; 35 dpi) were transcardially perfused with 0.1 M phosphate buffer (25 mM NaH_2_PO_4_•H_2_O; 75 mM Na_2_HPO_4_; pH 7.4) followed by 4% paraformaldehyde (PFA) in the same buffer. After extraction, the spinal cords were post-fixed in 4% paraformaldehyde for 4 h. Lumbar spinal cords were dissected, cryoprotected overnight in 20% sucrose at 4 ℃, embedded in Tissue-Tek OCT (Electron Microscopy Sciences, Hatfield, PA, USA), and stored at −80 ℃ until use. Tissue sections (10 μm-thick) were blocked for 60 min in TBS (100 mM Tris Base, 150 mM NaCl; pH 7.4) supplemented with 5% normal goat serum, 0.2% Triton X-100, and 3% Fab fragment (Jackson ImmunoResearch, West Grove, PA, USA) followed by overnight incubation at 4 ℃ with a primary antibodies against C3 (1:1500; Dako, Santa Clara, CA, USA; A0063) and GFAP (1:40; Millipore Burlington, MA, USA; MAB3402). Following extensive washing, primary antibodies were detected by incubation with appropriate Alexa Fluor 488 or 594 conjugated donkey antibodies (1:400; Invitrogen, Carlsbad, CA, USA) for 1 h at room temperature. Confocal microscopy of dual-labeled tissue sections was performed using a Leica TCS Sp8X STED CW confocal laser scanning microscope (Leica Microsystems, Wetzlar, Germany). A series of optical sections (z-stacks) was taken through the tissue at a spacing of 0.7 μm using a 20X oil immersion objective. White matter C3^+^ and GFAP^+^ stained areas were quantified in two optical sections per tissue slice obtained from 4–5 slices per mouse. Custom scripts were used for semiautomated image processing and analysis of co-localization in Fiji ImageJ [40].

### 2.6. Data Analysis

RT-qPCR analyses were carried out in 5–6 per experimental group. Immunohistochemistry was performed in 3 control and EAE mice. Data are presented as mean ± SEM and *n* represents the number of animals tested. Student’s *t*-tests or Mann–Whitney U-tests following application of Kolmogorov-Smirnov normality tests were used to determine significance in individual gene expression at different stages of EAE in comparison to naïve mice. One-way ANOVA followed by Newman–Keuls tests was used for the analysis of normalized gene expression changes over the disease course. Pearson correlation coefficients were calculated for Figures S3 and S4. All statistical comparisons were performed using the GraphPad Software with *p* < 0.05 considered statistically significant.

## 3. Results

### 3.1. Early Deregulation of Endocannabinoid System Genes in Astrocytes during EAE

**Figure 1:**
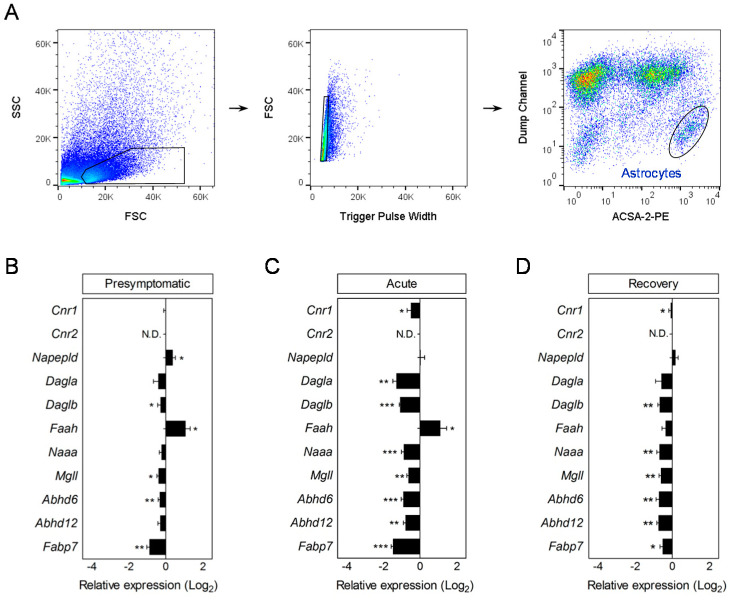
Early-onset deregulation of endocannabinoid system genes in astrocytes during EAE. (**A**) Representative dot-plots depicting FACS strategy for the purification of astrocytes from the CNS of EAE mice. Astrocytes were sorted as ACSA-2^+^ cells following the exclusion of contaminating microglia/macrophages and hematopoietic cells (CD11b^+^/CD45^+^), oligodendrocytes (O1^+^), oligodendrocyte progenitors (A2B5^+^), and endothelial cells (CD31^+^) grouped in a dump channel. (**B**–**D**) Histograms represent fold changes in the relative expression of endocannabinoid activity genes in cells sorted during presymptomatic (**B**), acute (**C**), and recovery (**D**) phases of the EAE compared to astrocytes from non-immunized mice purified in parallel (*n* = 5–6 mice group). Changes in relative expression were determined as log_2_. N.D., not detected. * *p* < 0.05; ** *p* < 0.01; *** *p* < 0.001; referred to naïve astrocytes by Student’s *t*-tests or Mann–Whitney *U*-tests.

To investigate the role of endocannabinoids in regulating astrocyte dysfunction in MS we analyzed the gene expression levels of the principal endocannabinoid signaling molecules in cells purified during EAE progression, using a microfluidic RT-qPCR screen. Interestingly, astrocytes isolated from asymptomatic disease showed significant modulation of several endocannabinoid system associated genes. In particular, we measured increased levels of *Napepld* together with a marked reduction in *Fabp7*, although these alterations were concomitant with augmented *Faah* transcripts (Figure 1B; Table S2). In addition, presymptomatic EAE astrocytes displayed down-regulated gene expression of the main 2-AG hydrolytic enzymes *Mgll* and *Abhd6* without changes in the α form of DAGL (*Dagla*), primary responsible of 2-AG synthesis in nervous tissue [41] (Figure 1B; Table S2).

Astrocytes isolated from acute EAE mice exhibited reductions in all endocannabinoid metabolism-associated genes, with the exception of *Faah*, which persisted at chronic disease (Figure 1C,D; Table S2). Blunted expression of endocannabinoid hydrolysis genes was associated with reduced *Cnr1* transcript levels both at acute and recovery phases. Cannabinoid CB_2_ receptor mRNA levels were not detected in astrocytes from naïve or EAE mice at any stage of disease progression (Figure 1C,D). This result is in general agreement with previous pharmacological studies in cultured cells that support only a marginal expression of CB_2_ receptors in astroglial cells [33]. On the other hand, we also measured down-regulated gene expression of *Dagla* and *Daglb* without changes in *Napepld*, suggesting a selective impairment of 2-AG biosynthetic molecules at the transcript level that parallels deficits in hydrolysis-related genes at acute and chronic disease stages (Figure 1C,D).

### 3.2. EAE Induction Promotes Astrocyte Conversion to a Neurotoxic A1 Phenotype

Recent tissue and cell-type-specific transcriptomic studies suggest a relevant role for neurotoxic A1 astrocytes in MS pathogenesis [42,43]. However, the induction pattern of astrocytic A1 phenotype related genes in rodent models of the disease remains to be fully characterized. To address this question, we evaluated the expression of A1 phenotype associated genes in astrocytes purified by flow cytometry at the different time-points of EAE progression. In parallel, we analyzed several genes associated with the protective A2 reactive phenotype, astrocyte pan-reactive genes, as well as genes involved in astrocyte functions during autoimmune demyelination [2,8,9,44].

**Figure 2:**
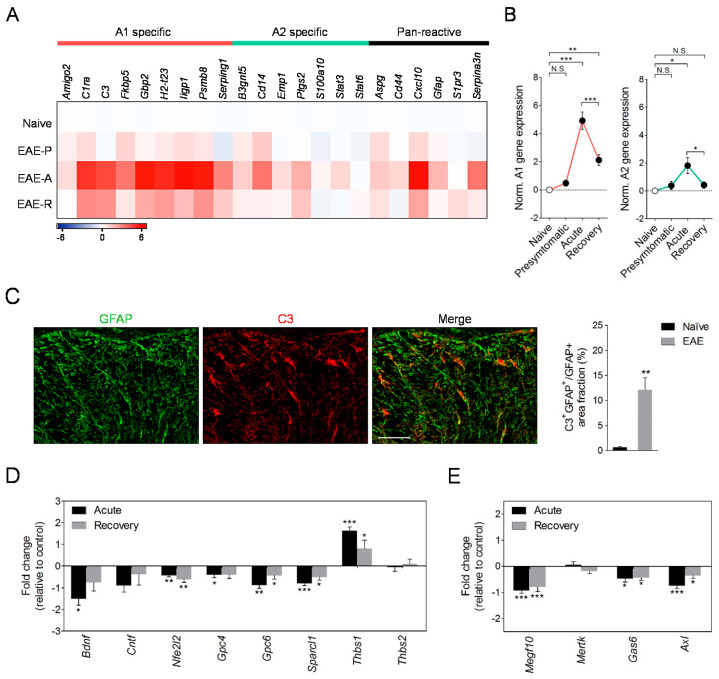
Modulation of astrocytic phenotype during EAE. (**A**) Heat map comparing the mean expression of neurotoxic A1, protective A2, and pan-reactive gene transcripts in astrocytes isolated from mice in the presymptomatic (EAE-P), acute (EAE-A) and recovery (EAE-R) phases of EAE, as determined by RT-qPCR (*n* = 5–6 mice group). (**B**) Histogram representation of normalized A1 and A2 gene expression during EAE progression. (**C**) Confocal images and quantification of complement component C3 colocalization with the astrocytic marker GFAP in spinal cord white matter of chronic EAE mice (*n* = 3; 35 dpi). Scale bar: 50 µm. (**D**,**E**) Fold change in mRNA expression of genes related to neuroprotection and synaptogenesis (**C**) and phagocytosis (**D**) in astrocytes sorted during acute and chronic EAE. Changes in relative expression were determined as log_2_. N.S., not statistically significant. * *p* < 0.05; ** *p* < 0.01; *** *p* < 0.001; referred to naïve astrocytes by Student´s *t*-tests or Mann–Whitney *U*-tests; one-way ANOVA followed by Newman–Keuls tests (**B**).

The results of our RT-qPCR screen show that astrocytes activated during EAE display increased levels of several pan-reactive genes (*Cxcl10*, *Gfap*, *Serpina3n*) and reduced mRNA expression of several astrocytic markers (*Aldh1/l1*, *Aqp4, Slc1a3*) (Figure 2A; Table S2), thus matching previous observations [43,45]. We also validated the up-regulation of genes encoding molecules involved in EAE, including *Ccl2*, *Ccl5*, and *Nos2*, and transcripts for genes of the interferon type I signaling pathway (*Stat1*, *Stat2*, *Irf9*, *Mx1*), at peak disease (Table S2) [8,45]. In addition, astrocytes markedly upregulated the mRNA expression of several A1 reactive genes at acute EAE with many of these molecules remaining elevated during the recovery phase (Figure 2A; Table S2). Remarkably, a number of A1 genes were significantly up-regulated in astrocytes purified as asymptomatic disease (Figure 2A; Table S2), suggesting that transformation of these cells towards a neurotoxic phenotype is an early event during EAE and parallels the infiltration of peripheral immune cells into the CNS (Figure S2). Interestingly, we found a highly significant correlation between neurological disability and up-regulation of *C3* transcript levels in astrocytes purified during acute EAE, but not in cells sorted at the recovery phase (Figure S3). However, deregulation of genes encoding additional A1 phenotype markers showed only non-significant trends or even inverse correlations with motor scores at acute or chronic disease stages (Figure S3). On the other hand, the expression of astrocytic A2 signature genes was either unchanged or only modestly increased during the time-course of EAE (Figure 2A; Table S2). Normalization analysis of A1 and A2 genes indicated a greater induction of the neurotoxic-marker genes during the time-course of EAE progression (Figure 2B).

To corroborate the presence of A1 astrocytes in EAE we evaluated the expression of C3 in spinal cord astrocytes at the protein level. Double immunolabeling using antibodies for complement component C3 [9] and the astrocytic marker GFAP showed increased expression of C3 in white matter astrocytes from chronic EAE mice compared to naïve animals (Figure 2C). Our colocalization analyses also demonstrate a predominant C3 expression by astrocytes in white matter of EAE mice as ~75% of immunostaining lied on the GFAP^+^ processes (Figure 2C). Since astrocytic C3 has been associated with a neurotoxic phenotype of aberrant synapse stripping and myelin phagocytic activity [9,46] we analyzed the gene expression of several molecules regulating these functions in cells purified during EAE. The induction of astrocytic A1 reactive transcripts at acute disease was paralleled by a significant down-regulation of genes involved in neuroprotection (*Bdnf*), synapse formation (*Gpc4*, *Gpc6, Sparcl1*) and phagocytosis (*Megf10*, *Gas6*, *Axl*) (Figure 1C,D). Cells purified at acute disease also displayed increased levels of the astrocytic synaptogenic molecule *Thbs1* in good agreement with the changes reported following the in vitro and in vivo induction of A1 astrocytes [9,10]. Most of these genes remained deregulated, albeit to a lesser extent, in astrocytes purified at the recovery phase of the EAE thus mirroring deregulation of A1 phenotype markers (Figure 1C,D; Table S2). Combined, these data show that astrocytes acquire neurotoxic A1 phenotype associated with deficiencies in maintaining and promoting synapses and blunted myelin clearance capacity during the time-course of EAE.

### 3.3. Modulation of Endocannabinoid Signaling-Related Genes in Microglia during EAE

**Figure 3:**
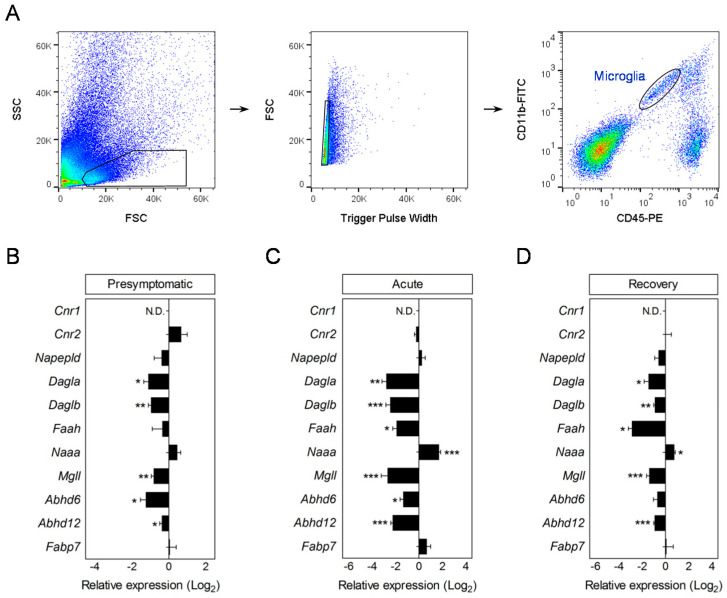
Altered expression of microglial endocannabinoid system genes during EAE. (**A**) Flow cytometry gating strategy for the analysis of microglial cells from EAE mice. Microglia were sorted as CD11b^+^ cells with low CD45 expression. (**B**–**D**) RT-qPCR analysis of endocannabinoid signaling genes in microglia purified at presymptomatic (**B**), acute (**C**), and recovery (**D**) phases of EAE. Fold changes in relative expression compared to naïve microglia were determined as log_2_ (*n* = 5–6 mice group). Changes in relative expression were determined as log_2_. N.D., not detected. * *p* < 0.05; ** *p* < 0.01; *** *p* < 0.001; referred to naïve astrocytes by Student´s *t*-tests or Mann–Whitney *U*-tests.

**We next assessed the expression level of endocannabinoid signaling-associated genes in microglia purified over the course of EAE.** In contrast to our observations in astrocytes, expression of *Cnr1* was not detected by RT-qPCR in naïve or EAE microglia. Conversely, *Cnr2* transcripts were expressed in cells from non-immunized mice but not significantly modulated over the course of EAE (Figure 3B–D; Table S3). Interestingly, microglia isolated from the CNS of EAE mice displayed changes in the expression levels of genes involved in 2-AG metabolism reminiscent to the ones observed in astrocytes during disease progression, albeit to a higher extent. In particular, our results show reductions in the transcript levels for *Mgll*, *Abhd6,* and *Abhd12* in microglia purified at asymptomatic disease, as well as at the peak of the symptoms (Figure 3B–D; Table S3). In parallel, microglial cells exhibited an early onset reduction in the expression of 2-AG production related genes *Dagla* and *Daglb*. Most of these transcriptional changes were present even though in lesser magnitude in microglial cells isolated from mice at the recovery phase of EAE (Figure 3D; Table S3). On the other hand, genes involved in AEA biosynthesis and metabolism were not significantly modulated in microglia at presymptomatic disease (Figure 3B; Table S3). Cells purified at acute and chronic disease stages, however, displayed a significant down-regulation of *Faah* paralleled by increased *Naaa* levels, without changes in the expression of *Napepld* or *Fabp7* (Figure 3B–D; Table S3). In sum, our results indicate that astrocytes and microglia differ in their transcriptional modulation of AEA signaling-related genes during EAE.

### 3.4. Expression Patterns of Pro-Inflammatory and Protective Genes in Microglia during EAE

**Figure 4:**
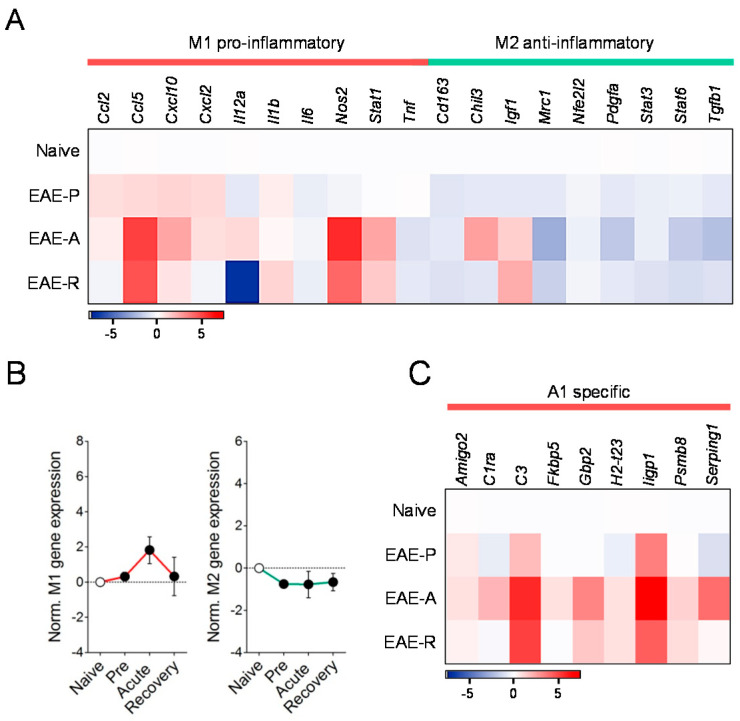
Pro-inflammatory and anti-inflammatory gene expressions in microglia isolated during EAE. (**A**) Heat map depicting the mean expression of M1 and M2 phenotype associated genes in microglial cells purified from mice at presymptomatic (EAE-P), acute (EAE-A) and recovery (EAE-R) stages of EAE as determined by RT-qPCR. (**B**) Analysis of normalized gene expression by one-way ANOVA determined no overall deregulation of M1 or M2 gene transcripts during disease progression. (**C**) Mean expression of A1 neurotoxic genes in microglial cells during the time-course of EAE. Fold changes in the relative expression compared to naïve microglia were determined as log_2_ (*n* = 5–6 mice group) Pre, Presymptomatic. N.S., not statistically significant. referred to microglia purified from naïve mice by Student´s *t*-tests or Mann–Whitney *U*-tests.

The activation of microglia in MS has been described as pro-inflammatory (also known as M1) and regenerating (regarded as M2) states [5,47] although more recent observations suggest a multidimensional model of activation [48]. Concerning findings during autoimmune demyelination, RNA sequencing of microglia from EAE mice shows a changing phenotype over the course of the disease with an early-onset modulation of chemokines and molecules related to apoptotic cell uptake [49]. Consistent with these results, a number of genes associated with the induction and expression of pro-inflammatory responses by microglia such as *Ccl2*, *Ccl5*, *Cxcl2*, *Nos2*, or *Stat1* displayed upregulated levels in cells purified from EAE mice, as determined by RT-qPCR (Figure 4A; Table S3). Although the induction of M1 phenotype genes was most remarkable at acute disease, some of these molecules were already upregulated in microglia isolated from animals at presymptomatic EAE (Figure 4A; Table S3), thus paralleling the early induction of A1 genes in astrocytes. These results sit well with the hypothesis that microglia activated during CNS disease facilitates the induction of a neurotoxic phenotype in astrocytes in vivo [9]. It is also noteworthy, however, that some pro-inflammatory genes were either not modulated (*Il6*), or even down-regulated (*Il12a*, *Tnfa*), in microglia purified during EAE. On the other hand, most microglia M2 phenotype related genes displayed reduced expression (Figure 4A; Table S3). Despite our observations regarding changes in the transcript levels of individual genes suggest that microglia in EAE predominantly display pro-inflammatory characteristics, normalization analysis showed no overall modulation of the M1 and M2 associated genes tested during the time-course of the disease (Figure 4B). Further, none of the M1 genes tested showed a significant correlation with disease scores at acute or chronic EAE (data not shown). These results contrast the marked induction of A1 genes in astrocytes (Figure 1A) and match previous reports showing that a major subset of microglial cells exhibits an intermediate activation state during autoimmune demyelination [50,51].

### 3.5. EAE Induces the Expression of Astrocyte Pan-Reactive and A1-Like Genes in Microglia

According to recent reports, CNS disease conditions such as spinal cord injury and amyotrophic lateral sclerosis induce an autologous expression of classical astrocyte-specific marker genes in microglia whose biological role is currently unknown [52,53]. To understand if such processes are occurring in MS, we assessed the mRNA levels of pan-astrocytic genes and A1 phenotype markers in microglial cells purified during EAE. Consistent with previous observations [52], microglia from EAE mice at acute disease over-expressed markers of reactive astrogliosis such as *Gfap*, *Serpina3n*, *S1pr3*, and *Cd44* (Table S3). Furthermore, transcriptomic responses of microglial cells during EAE mirrored the upregulated expression of A1 genes in astrocytes, with increased levels of complement components *C1ra* and *C3*, among other phenotype markers (Figure 4C; Table S3). These findings indicate that the induction of genes associated with the expression of a neurotoxic astrocyte phenotype is not exclusive to this cell population during EAE. However, the deregulation of A1-like genes in microglia did not significantly correlate with the severity of disease scores at acute or chronic EAE disease (Figure S4). In all, these results argue against the pathological relevance of A1 gene expression in microglial cells during EAE.

## 4. Discussion

The study of factors and mechanisms regulating astrocyte and microglia activation in MS has important therapeutic implications and is a matter of intense research. Findings of the current study indicate that these cell populations undergo multiple transcriptomic alterations affecting endocannabinoid system genes concomitant to their phenotypic activation during EAE and that these alterations occur early after disease induction.

A role for endocannabinoids as modulators of MS pathology is supported by a number of studies reporting altered levels of these lipids in patients and rodent models of the disease. Increased concentrations of AEA have been determined in brain tissue, cerebrospinal fluid, and plasma from MS patients [28,29,30,31]. These observations have been replicated in EAE and correlated to increased NAPE-PLD and reduced FAAH expression and activities [30,32]. Concerning 2-AG, the majority of the studies report unaltered concentrations both in animal models and MS patients [21,24,30], although higher 2-AG levels have also been proposed in EAE mice [32]. Although it is generally assumed that changes in endocannabinoid levels may be, at least in part, associated to the inflammatory reaction driven by innate immune cells in MS tissue, whether the transcriptional reprogramming of astrocytes and microglia during disease progression involves changes in the endocannabinoid biosynthetic and metabolic machinery has never been assessed. In this study, we show that both cell populations display significant reductions in 2-AG hydrolysis genes *Mgll*, *Abhd6*, and *Abhd12* at the early stages of EAE, paralleled or followed by reductions in *Dagla* and *Daglb* transcripts. Altogether, these results suggest that time-dependent transcriptional changes in astrocytes and microglial cells may contribute to the dysregulation of 2-AG levels in MS, and help explain some inconsistencies between previous studies. On the other hand, astrocytes and microglia purified over the course of EAE differ in the timing, extent, and sign of transcriptional changes affecting AEA production and metabolism machinery. Astroglia transiently and selectively over-expressed *Napepld* transcripts at presymptomatic disease while displaying early and persistent down-regulation of *Naaa* and *Fabp7* expression levels. However, astrocytes at asymptomatic and acute EAE also displayed increased transcript levels for *Faah*, consistent with the predominant localization of FAAH proteins in hypertrophic astroglia associated to active plaques in human MS [54]. Conversely, isolated microglial cells showed selective down-regulation of *Faah* transcripts paralleled by increased *Naaa* expression levels at acute and chronic stages. In sum, these findings illustrate the cell-type specificity of transcriptomic alterations regarding AEA biosynthesis and hydrolysis genes in a demyelinating context and suggest that both astroglia and microglial cells participate in the misbalance of AEA levels in MS.

A hurdle when interpreting the biological impact of changes in the gene expression pattern of endocannabinoid synthetic and metabolic machinery in purified cells is the absence of results showing parallel modifications in 2-AG and AEA levels. In this study, the low number of astrocytes and microglial cells purified for analysis precluded us from providing experimental evidence of changes in endocannabinoid contents. In this regard, the development of high-sensitivity liquid chromatography-mass spectrometry techniques allows nowadays the detection of very low amounts of endocannabinoids, but this experimental approach requires a >10^6^ cells for accurate and reliable detection of changes in AEA and 2-AG levels [55,56,57,58]. When evaluating the utility of endocannabinoid measurements as a readout of unbalanced production/metabolism it should also be kept in mind that these alterations may be activity-dependent in vivo and, therefore, not faithfully mirrored upon determination of basal levels in purified cells. Experimental limitations concerning cell availability in this study also applied to the detection of proteins for the main components of the endocannabinoid system, which is further hindered by the low expression levels of several of these molecules in astrocytes and microglia as well as by antibody specificity issues [59,60,61]. Although the outcome of expression changes in the production and metabolic genes on endocannabinoid levels in the vicinity of the cells remains, thus, to be established, gene expression results in astrocytes purified at asymptomatic EAE lead us to postulate an imbalance in 2-AG mediated activity at early disease stages. Indeed, down-regulated *Mgll* and *Abhd6* transcript levels in astrocytes without concomitant reductions in *Dagla* expression may be associated with enhanced 2-AG levels in the context of previous work. This possibility is supported by lipase profiling experiments demonstrating that MAGL and ABHD6 are responsible for approximately 85% and 8% of brain 2-AG hydrolysis [14], as well as by results from global pharmacological or genetic inactivation studies showing that both enzymes control the accumulation and biological activity of this lipid mediator [17,56]. Further, the analysis of astrocyte-specific MAGL knockout mice has consistently demonstrated that astrocytic MAGL plays a substantive role in 2-AG metabolism its inactivation leading to deficient endocannabinoid clearance. In particular, conditional deletion of MAGL in astrocytes leads to a 40% reduction in brain 2-AG hydrolysis and 3-fold increases in 2-AG levels while limiting the spatial diffusion of endocannabinoid signaling at neuronal CB_1_ receptors [62,63,64]. On the other hand, there is also consensus that MAGL inactivation triggers CB_1_ receptor down-regulation upon sustained exposure to increased endocannabinoid levels [20,65]. Consistently, astrocytes purified during acute, but not asymptomatic, EAE disease showed reduced *Cnr1* transcript levels that persisted at the recovery phase. Although changes in endocannabinoid levels cannot be inferred based only on transcriptional deregulation and caution is thus needed when interpreting these data, altogether our results point to an early shift toward enhanced 2-AG mediated activity onto astrocytic CB_1_ receptors that may be followed by deficits at more progressive stages of the autoimmune demyelination process.

Our data concerning deregulation of *Mgll*, *Abhd6*, *Dagla*, *Faah,* and *Fabp7* transcript levels in astrocytes purified during acute EAE confirm results from a recent transcriptomic study in EAE mice at 45 days post-immunization [43]. These authors used RiboTag technology applied to Gfap-Cre mice to determine astrocyte-specific gene expression networks in multiple regions of the CNS but did not validate RNA-seq results using RT-qPCR and did not report changes in additional endocannabinoid system genes. It is noteworthy, however, that two studies using fundamentally different experimental approaches show similar alterations regarding endocannabinoid activity associated genes at acute/chronic EAE stages. Altogether, these observations indicate that deregulated astrocytic expression of endocannabinoid biosynthesis and metabolism genes is a consistent finding in the EAE mouse model of MS.

With regard to the activation characteristics of the astroglia population in EAE, this study reports that CNS astrocytes purified during the time-course of the disease dynamically and highly up-regulate A1-like genes as compared to A2 phenotype markers. We establish that astroglial cells over-express a number of A1 reactive genes at early, asymptomatic stages, with most of these molecules exhibiting significantly increased transcript levels both at acute and recovery phases. Using immunohistochemistry, we corroborated that a population of reactive astrocytes in spinal cord white matter up-regulates complement component C3 at the protein level. Consistent with the acquisition of pathogenic functions, our RT-qPCR analysis also demonstrates that induction of astrocytic A1 phenotype marker genes during autoimmune demyelination is associated with the deregulation of specific genes involved in neuroprotection, synaptogenesis, and phagocytosis, thus matching previous observations [9,10]. These observations add to the growing body of evidence showing the presence of a population of neurotoxic A1 astrocytes in brain aging and chronic neurodegenerative diseases that compromise synaptic function in part through the release of C3 [46,66,67,68]. Furthermore, in this study, the increase in astrocytic *C3* transcripts strongly correlated with a motor disability at the acute disease. Although neurological disability in a complex disease model such as EAE cannot be ascribed to variations in a single gene, this result is reminiscent of the negative correlation between astroglial C3 expression and retinal ganglion cell density in optic neuritis [10], and lead us to postulate a relevant connection between astrocytic complement expression in white matter tracts and neurodegeneration in MS. On the other hand, the transcriptional induction of additional astrocytic A1 markers displayed, however, a complex profile in terms of correlation with disease severity, with many of them showing non-significant correlations or even inversely correlating with neurological scores. When interpreting these results, it should be kept in mind that although the general up-regulation of A1 genes reported here supports the expression of a pathogenic neurotoxic astrocytic phenotype [9,44,67] each one of these individual genes might play opposite roles in regulating astrocyte function in terms of disease outcome. Indeed, the inverse correlation astrocytic IFNγ inducible genes such as *Iigp1* and *Psmb8* and disease severity is not surprising in the context of protective responses driven by this signaling pathway in experimental MS [69,70]. In addition, most of the A1-like genes assayed in this study are not expected to be exclusively expressed in the population of so-called neurotoxic astrocytes during EAE. Instead, a complex pattern of expression in several astrocyte populations displaying different polarization states may be suggested for the different genes tested based on the existing literature. Importantly, single-cell microfluidic qPCR of astrocytes purified from experimental models for astrocyte polarization have validated *C3* among other A1 genes as the most appropriate marker to visualize the population of neurotoxic astrocytes in disease conditions, based on its selective expression in this subset of reactive astroglia [9]. In this scenario, our results corroborate the pathological relevance of A1 astrocytes expressing C3 during autoimmune demyelination and highlight the complexity of studying astrocyte phenotypic responses in MS.

Microfluidic RT-qPCR analysis of isolated microglia revealed a preferential activation toward a pro-inflammatory phenotype with increased expressions of chemokines such as *Ccl2*, *Ccl5*, *Cxcl2*, and *Cxcl10* that can promote hematopoietic cell recruitment, hence matching previous transcriptomic analyses [49]. However, it is also noteworthy that the induction of these injury-promoting molecules was paralleled by the overexpression of genes involved in the expression of microglia protective functions such as *Igf1* and *Chil3*. In this regard, our observations add to the growing body of evidence indicating that microglial cells acquire complex phenotypes during autoimmune demyelination that may promote both tissue damage and repair [51,71]. On the other hand, we also demonstrate the induction of genes associated with reactive astrogliosis and neurotoxic A1 functions in microglia during EAE. The expression of microglial *Gfap* and *Serpina3n* transcripts shown in this study has been previously reported in mouse models of injury and disease, and it is speculated that it might suppress pro-inflammatory pathways [52,53]. Our results in the EAE model support additional studies using cell-type-specific transcriptomic analysis of microglia and complementary in vitro analyses to uncover the precise role of astrocyte population marker genes within these cells in MS pathophysiology. With regard to astrocytic A1-like genes, overexpression of *C1ra* and *C3* is in line with previous results from transcriptomic studies demonstrating the up-regulation of the complement pathway in microglia and monocyte-derived macrophages purified from EAE mice [49]. A causal role of complement in the context of demyelination has been established based on studies showing that C3 null mice display attenuated disease severity in the EAE model [72]. However, the correlation between astrocytic *C3* induction and acute EAE severity in this study was not detected in microglia purified in parallel from the same animals, and immunohistochemical analysis demonstrated that the expression of this A1 population marker is predominantly associated to astrocytic profiles in EAE mice. These observations provide evidence that astrocytes, and not microglial cells, are the main mediators of pathogenic C3 signaling in EAE and thus confer cell-type specificity to the protective phenotype C3 knockout mice in this MS mouse model.

A role for endocannabinoids in preventing or attenuating reactive astrogliosis and microglial reaction in MS mouse models is supported by a number of studies using genetic and pharmacological tools [19,20,23,24]. However, how these observations translate to disease progression in terms of pathogenic and protective astrocyte and microglia functions and the detailed mechanisms underlying such effects remain to be fully elucidated. The early deregulation of endocannabinoid system genes in astrocytic and microglia during EAE reported here supports the possibility that these lipid mediators tune the phenotypic transformation of these cells in demyelinating conditions and warrants further investigation. In the same context, the extent to which alterations in the expression pattern of endocannabinoid system genes in innate immune cells determines the general neuroprotective and regenerative role of these lipids in MS requires further analysis. In this regard, a role for astroglial and microglial endocannabinoid systems in neurodegenerative conditions is supported by accumulating evidence demonstrating the relevance of endocannabinoid-mediated crosstalk mechanisms between innate immune cells and neurons in regulating brain functions [73,74]. Concerning endocannabinoid signaling in MS, neuronal CB_1_ receptors and CB_2_ receptors in T cells have been established as crucial determinants of cannabinoid-mediated EAE suppression based, in part, on studies using cell-type-specific knockdown strategies [24]. Similar experimental approaches have not yet been applied to the analysis of the neuroglial endocannabinoid system in MS likely due to the low expression levels of cannabinoid receptor proteins in these cells. In the light of these results, further studies using cell-type-specific transgenic mice or virus-mediated strategies to specifically modulate endocannabinoid system elements in astrocytes and microglia are needed to decipher the relevance of these cell types as targets and mediators of endocannabinoid signaling in MS.

## 5. Conclusions

Our data represent the first RT-qPCR analysis of endocannabinoid system genes in astrocytes and microglia at multiple time-points of autoimmune demyelination and disease severity. Results show that activation of innate immune cells in MS is associated with both general and cell-type-specific transcriptional alterations affecting endocannabinoid activity associated molecules. These adaptations are time-dependent, with an early and progressive down-regulation of genes encoding 2-AG hydrolytic enzymes that precede the appearance of neurological disability followed or paralleled by reductions in transcripts for cannabinoid receptors and synthetic endocannabinoid enzymes. Concerning the phenotype of innate immune cells during the disease course, we confirm that microglia exhibit an intermediate polarization state regarding the expression of inflammatory genes during EAE. Finally, we demonstrate disease-induced over-expression of astrocytic A1-like phenotype genes paralleled by deficits in synaptic protection and phagocytosis related transcripts and support a pathogenic role of this subset of reactive astrocytes in MS.

## Supplementary Materials

The following are available online at https://www.mdpi.com/2218-273X/10/9/1228/s1, Figure S1: FACS sorting and purity of CNS cell populations during EAE. (**A**,**B**) Representative dot-plots depicting FACS strategy for the purification of astrocytes and microglia from EAE mice. Astrocytes were sorted as ACSA-2+ cells following exclusion of contaminating hematopoietic cells (CD45+), microglia/macrophages, and hematopoietic cells (CD11b+/CD45+), oligodendrocytes (O1+), oligodendrocyte progenitors (A2B5+) and endothelial cells (CD31+) grouped in a dump channel. *Right*: The purity of sorted astrocyte population by FACS analysis of the astrocytic marker GLAST. (**B**) Microglial cells were sorted as CD11b positive cells with low CD45 expression (CD11b+/CD45low). (**C**) qPCR analysis of astrocyte (*Aldh1l1*, *Aqp4*, *Atp1b2*, *Gfap*, *Slc1a3*) and microglia (*Gpr34*, *Itgam*, *P2ry12*, *Tmem119*) enriched genes in both cell types purified at acute EAE disease (*n* = 5–6 mice; normalized to astrocytic *Gfap*)., Figure S2: Cell-population analysis during EAE. (**A**) Clinical score of the diseased animals used in the study (*n* = 19 mice; 2 independent EAE experiments). Astrocytes and microglia were sorted from control and immunized mice at the pre-symptomatic (7–8 dpi), acute (14–17 dpi) and recovery (28–31 dpi). (**B**) Neurological score of the diseased mice used for FACS analysis at each time-point (*n* = 6 mice). ****p* < 0.001; one-way ANOVA followed by Newman–Keuls tests. (**C**) Relative abundance of CD11b+CD45high and CD11b-CD45high peripheral immune cell populations in CNS single cell suspensions from control and EAE mice at pre-symptomatic, acute, and recovery phases of the disease (*n* = 6–8 mice per condition). ****p* < 0.001 referred to CD11b+ cells, Student´s *t*-tests., Table S1: Target genes and primers for RT-qPCR analysis in astrocytes and microglia, Table S2: RT-qPCR analysis of astrocytes purified during EAE versus control cells, Table S3: RT-qPCR analysis of microglia purified during EAE versus control cells.

## Abbreviations

N-acylethanolamine acid amidase (NAAA); 2-arachidonoylethanolamide (anandamide, AEA); N-arachidonoylethanolamine phospholipase D (NAPE-PLD); 2-arachidonoylglycerol (2-AG); diacylglycerol lipase α isoform (DGLα); diacylglycerol lipase β isoform (DGLα); experimental autoimmune encephalomyelitis (EAE); fatty acid amide hydrolase (FAAH); fatty acid binding protein subtype 7 (FABP7); α/β-hydrolase domain containing 6 (ABHD6); α/β-hydrolase domain containing 12 (ABHD12); monoacylglycerol lipase (MAGL); retinal ganglion cell (RGC).

## References

[1] Nylander A., Hafler D.A. (2012). Multiple sclerosis. J. Clin. Investig..

[2] Mayo L., Trauger S.A., Blain M., Nadeau M., Patel B., Alvarez J.I., Mascanfroni I.D., Yeste A., Kivisäkk P., Kallas K. (2014). Regulation of astrocyte activation by glycolipids drives chronic CNS inflammation. Nat. Med..

[3] Mayo L., Quintana F.J., Weiner H.L. (2012). The innate immune system in demyelinating disease. Immunol. Rev..

[4] Rothhammer V., Borucki D.M., Tjon E.C., Takenaka M.C., Chao C.C., Ardura-Fabregat A., de Lima K.A., Gutiérrez-Vázquez C., Hewson P., Staszewski O. (2018). Microglial control of astrocytes in response to microbial metabolites. Nature.

[5] Chu F., Shi M., Zheng C., Shen D., Zhu J., Zheng X., Cui L. (2018). The roles of macrophages and microglia in multiple sclerosis and experimental autoimmune encephalomyelitis. J. Neuroimmunol..

[6] Voet S., Prinz M., van Loo G. (2018). Microglia in central nervous system inflammation and multiple sclerosis pathology. Trends Mol. Med..

[7] Miron V.E., Franklin R.J. (2014). Macrophages and CNS remyelination. J. Neurochem..

[8] Rothhammer V., Mascanfroni I.D., Bunse L., Takenaka M.C., Kenison J.E., Mayo L., Chao C.C., Patel B., Yan R., Blain M. (2016). Type I interferons and microbial metabolites of tryptophan modulate astrocyte activity and central nervous system inflammation via the aryl hydrocarbon receptor. Nat. Med..

[9] Liddelow S.A., Guttenplan K.A., Clarke L.E., Bennett F.C., Bohlen C.J., Schirmer L., Bennett M.L., Münch A.E., Chung W.S., Peterson T.C. (2017). Neurotoxic reactive astrocytes are induced by activated microglia. Nature.

[10] Tassoni A., Farkhondeh V., Itoh Y., Itoh N., Sofroniew M.V., Voskuhl R.R. (2019). The astrocyte transcriptome in EAE optic neuritis shows complement activation and reveals a sex difference in astrocytic C3 expression. Sci. Rep..

[11] Hou B., Zhang Y., Liang P., He Y., Peng B., Liu W., Han S., Yin J., He X. (2020). Inhibition of the NLRP3-inflammasome prevents cognitive deficits in experimental autoimmune encephalomyelitis mice via the alteration of astrocyte phenotype. Cell Death Dis..

[12] Iannotti F.A., Di Marzo V., Petrosino S. (2016). Endocannabinoids and endocannabinoid-related mediators: Targets, metabolism and role in neurological disorders. Prog Lipid Res..

[13] Ahn K., McKinney M.K., Cravatt B.F. (2008). Enzymatic pathways that regulate endocannabinoid signaling in the nervous system. Chem. Rev..

[14] Blankman J.L., Simon G.M., Cravatt B.F. (2007). A comprehensive profile of brain enzymes that hydrolyze the endocannabinoid 2-arachidonoylglycerol. Chem. Biol..

[15] Kaczocha M., Glaser S.T., Deutsch D.G. (2009). Identification of intracellular carriers for the endocannabinoid anandamide. Proc. Natl. Acad. Sci. USA.

[16] Cravatt B.F., Demarest K., Patricelli M.P., Bracey M.H., Giang D.K., Martin B.R., Lichtman A.H. (2001). Supersensitivity to anandamide and enhanced endogenous cannabinoid signaling in mice lacking fatty acid amide hydrolase. Proc. Natl. Acad. Sci. USA.

[17] Long J.Z., Li W., Booker L., Burston J.J., Kinsey S.G., Schlosburg J.E., Pavón F.J., Serrano A.M., Selley D.E., Parsons L.H. (2009). Selective blockade of 2-arachidonoylglycerol hydrolysis produces cannabinoid behavioral effects. Nat. Chem. Biol..

[18] Kaczocha M., Glaser S.T., Maher T., Clavin B., Hamilton J., O’Rourke J., Rebecchi M., Puopolo M., Owada Y., Thanos P.K. (2015). Fatty acid binding protein deletion suppresses inflammatory pain through endocannabinoid/N-acylethanolamine-dependent mechanisms. Mol. Pain.

[19] Ortega-Gutiérrez S., Molina-Holgado E., Arévalo-Martín A., Correa F., Viso A., López-Rodríguez M.L., Di Marzo V., Guaza C. (2005). Activation of the endocannabinoid system as therapeutic approach in a murine model of multiple sclerosis. FASEB J..

[20] Bernal-Chico A., Canedo M., Manterola A., Victoria Sánchez-Gómez M., Pérez-Samartín A., Rodríguez-Puertas R., Matute C., Mato S. (2015). Blockade of monoacylglycerol lipase inhibits oligodendrocyte excitotoxicity and prevents demyelination in vivo. Glia.

[21] Manterola A., Bernal-Chico A., Cipriani R., Canedo-Antelo M., Moreno-García Á., Martín-Fontecha M., Pérez-Cerdá F., Sánchez-Gómez M.V., Ortega-Gutiérrez S., Brown J.M. (2018). Deregulation of the endocannabinoid system and therapeutic potential of ABHD6 blockade in the cuprizone model of demyelination. Biochem. Pharmacol..

[22] Arévalo-Martín A., Vela J.M., Molina-Holgado E., Borrell J., Guaza C. (2003). Therapeutic action of cannabinoids in a murine model of multiple sclerosis. J. Neurosci..

[23] Palazuelos J., Davoust N., Julien B., Hatterer E., Aguado T., Mechoulam R., Benito C., Romero J., Silva A., Guzmán M. (2008). The CB(2) cannabinoid receptor controls myeloid progenitor trafficking: Involvement in the pathogenesis of an animal model of multiple sclerosis. J. Biol. Chem..

[24] Maresz K., Pryce G., Ponomarev E.D., Marsicano G., Croxford J.L., Shriver L.P., Ledent C., Cheng X., Carrier E.J., Mann M.K. (2007). Direct suppression of CNS autoimmune inflammation via the cannabinoid receptor CB1 on neurons and CB2 on autoreactive T cells. Nat. Med..

[25] Berrendero F., Sánchez A., Cabranes A., Puerta C., Ramos J.A., García-Merino A., Fernández-Ruiz J. (2001). Changes in cannabinoid CB(1) receptors in striatal and cortical regions of rats with experimental allergic encephalomyelitis, an animal model of multiple sclerosis. Synapse.

[26] Cabranes A., Pryce G., Baker D., Fernández-Ruiz J. (2006). Changes in CB1 receptors in motor-related brain structures of chronic relapsing experimental allergic encephalomyelitis mice. Brain Res..

[27] Loría F., Petrosino S., Mestre L., Spagnolo A., Correa F., Hernangómez M., Guaza C., Di Marzo V., Docagne F. (2008). Study of the regulation of the endocannabinoid system in a virus model of multiple sclerosis reveals a therapeutic effect of palmitoylethanolamide. Eur. J. Neurosci..

[28] Jean-Gilles L., Feng S., Tench C.R., Chapman V., Kendall D.A., Barrett D.A., Constantinescu C.S. (2009). Plasma endocannabinoid levels in multiple sclerosis. J. Neurol. Sci..

[29] Di Filippo M., Pini L.A., Pelliccioli G.P., Calabresi P., Sarchielli P. (2008). Abnormalities in the cerebrospinal fluid levels of endocannabinoids in multiple sclerosis. J. Neurol. Neurosurg. Psychiatry..

[30] Centonze D., Bari M., Rossi S., Prosperetti C., Furlan R., Fezza F., De Chiara V., Battistini L., Bernardi G., Bernardini S. (2007). The endocannabinoid system is dysregulated in multiple sclerosis and in experimental autoimmune encephalomyelitis. Brain.

[31] Eljaschewitsch E., Witting A., Mawrin C., Lee T., Schmidt P.M., Wolf S., Hoertnagl H., Raine C.S., Schneider-Stock R., Nitsch R. (2006). The endocannabinoid anandamide protects neurons during CNS inflammation by induction of MKP-1 in microglial cells. Neuron.

[32] Baker D., Pryce G., Croxford J.L., Brown P., Pertwee R.G., Makriyannis A., Khanolkar A., Layward L., Fezza F., Bisogno T. (2001). Endocannabinoids control spasticity in a multiple sclerosis model. FASEB J..

[33] Stella N. (2010). Cannabinoid and cannabinoid-like receptors in microglia, astrocytes, and astrocytomas. Glia.

[34] Lutz B., Marsicano G., Maldonado R., Hillard C.J. (2015). The endocannabinoid system in guarding against fear, anxiety and stress. Nat. Rev. Neurosci..

[35] Batiuk M.Y., De Vin F., Duqué S.I., Li C., Saito T., Saido T., Fiers M., Belgard T.G., Holt M.G. (2017). An immunoaffinity-based method for isolating ultrapure adult astrocytes based on ATP1B2 targeting by the ACSA-2 antibody. J. Biol. Chem..

[36] Kantzer C.G., Boutin C., Herzig I.D., Wittwer C., Reiß S., Tiveron M.C., Drewes J., Rockel T.D., Ohlig S., Ninkovic J. (2017). Anti-ACSA-2 defines a novel monoclonal antibody for prospective isolation of living neonatal and adult astrocytes. Glia.

[37] Szulzewsky F., Pelz A., Feng X., Synowitz M., Markovic D., Langmann T., Holtman I.R., Wang X., Eggen B.J., Boddeke H.W. (2015). Glioma-associated microglia/macrophages display an expression profile different from M1 and M2 polarization and highly express Gpnmb and Spp1. PLoS ONE.

[38] Rothstein J.D., Martin L., Levey A.I., Dykes-Hoberg M., Jin L., Wu D., Nash N., Kuncl R.W. (1994). Localization of neuronal and glial glutamate transporters. Neuron.

[39] Jang J.S., Simon V.A., Feddersen R.M., Rakhshan F., Schultz D.A., Zschunke M.A., Lingle W.L., Kolbert C.P., Jen J. (2011). Quantitative miRNA expression analysis using fluidigm microfluidics dynamic arrays. BMC Genomics.

[40] Schindelin J., Arganda-Carreras I., Frise E., Kaynig V., Longair M., Pietzsch T., Preibisch S., Rueden C., Saalfeld S., Schmid B. (2012). Fiji: An open-source platform for biological-image analysis. Nat. Methods.

[41] Reisenberg M., Singh P.K., Williams G., Doherty P. (2012). The diacylglycerol lipases: Structure, regulation and roles in and beyond endocannabinoid signaling. Philos. Trans. R. Soc. Lond. B Biol. Sci..

[42] Zhao Y., Yamasaki R., Yamaguchi H., Nagata S., Une H., Cui Y., Masaki K., Nakamuta Y., Iinuma K., Watanabe M. (2020). Oligodendroglial connexin 47 regulates neuroinflammation upon autoimmune demyelination in a novel mouse model of multiple sclerosis. Proc. Natl. Acad. Sci. USA.

[43] Itoh N., Itoh Y., Tassoni A., Ren E., Kaito M., Ohno A., Ao Y., Farkhondeh V., Johnsonbaugh H., Burda J. (2018). Cell-specific and region-specific transcriptomics in the multiple sclerosis model: Focus on astrocytes. Proc. Natl. Acad. Sci. USA.

[44] Zamanian J.L., Xu L., Foo L.C., Nouri N., Zhou L., Giffard R.G., Barres B.A. (2012). Genomic analysis of reactive astrogliosis. J. Neurosci..

[45] Mayo L., Da Cunha A.P., Madi A., Beynon V., Yang Z., Alvarez J.I., Prat A., Sobel R.A., Kobzik L., Lassmann H. (2016). IL-10-dependent Tr1 cells attenuate astrocyte activation and ameliorate chronic central nervous system inflammation. Brain.

[46] Lian H., Yang L., Cole A., Sun L., Chiang A.C.-A., Fowler S.W., Shim D.J., Rodriguez-Rivera J., Taglialatela G., Jankowsky J.L. (2015). NFκB-activated astroglial release of complement C3 compromises neuronal morphology and function associated with Alzheimer’s disease. Neuron.

[47] Hu X., Leak R.K., Shi Y., Suenaga J., Gao Y., Zheng P., Chen J. (2015). Microglial and macrophage polarization—New prospects for brain repair. Nat. Rev. Neurol..

[48] Ransohoff R.M. (2016). A polarizing question: Do M1 and M2 microglia exist?. Nat. Neurosci..

[49] Lewis N.D., Hill J.D., Juchem K.W., Stefanopoulos D.E., Modis L.K. (2014). RNA sequencing of microglia and monocyte-derived macrophages from mice with experimental autoimmune encephalomyelitis illustrates a changing phenotype with disease course. J. Neuroimmunol..

[50] Zabala A., Vazquez-Villoldo N., Rissiek B., Gejo J., Martin A., Palomino A., Perez-Samartín A., Pulagam K.R., Lukowiak M., Capetillo-Zarate E. (2018). P2X4 receptor controls microglia activation and favors remyelination in autoimmune encephalitis. EMBO Mol. Med..

[51] Vogel D.Y.S., Vereyken E.J.F., Glim J.E., Heijnen P.D.A.M., Moeton M., van der Valk P., Amor S., Teunissen C.E., van Horssen J., Dijkstra C.D. (2013). Macrophages in inflammatory multiple sclerosis lesions have an intermediate activation status. J. Neuroinflammation.

[52] Noristani H.N., Gerber Y.N., Sabourin J.C., Le Corre M., Lonjon N., Mestre-Frances N., Hirbec H.E., Perrin F.E. (2017). RNA-Seq Analysis of microglia reveals time-dependent activation of specific genetic programs following spinal cord injury. Front. Mol. Neurosci..

[53] Chiu I.M., Morimoto E.T.A., Goodarzi H., Liao J.T., O’Keeffe S., Phatnani H.P., Muratet M., Carroll M.C., Levy S., Tavazoie S. (2013). A neurodegeneration-specific gene-expression signature of acutely isolated microglia from an amyotrophic lateral sclerosis mouse model. Cell Rep..

[54] Benito C., Romero J.P., Tolón R.M., Clemente D., Docagne F., Hillard C.J., Guaza C., Romero J. (2007). Cannabinoid CB1 and CB2 receptors and fatty acid amide hydrolase are specific markers of plaque cell subtypes in human multiple sclerosis. J. Neurosci..

[55] Muccioli G.G., Xu C., Odah E., Cudaback E., Cisneros J.A., Lambert D.M., López Rodríguez M.L., Bajjalieh S., Stella N. (2007). Identification of a novel endocannabinoid-hydrolyzing enzyme expressed by microglial cells. J. Neurosci..

[56] Marrs W.R., Blankman J.L., Horne E.A., Thomazeau A., Lin Y.H., Coy J., Bodor A.L., Muccioli G.G., Hu S.S., Woodruff G. (2010). The serine hydrolase ABHD6 controls the accumulation and efficacy of 2-AG at cannabinoid receptors. Nat. Neurosci..

[57] Hermes D.J., Xu C., Poklis J.L., Niphakis M.J., Cravatt B.F., Mackie K., Lichtman A.H., Ignatowska-Jankowska B.M., Fitting S. (2018). Neuroprotective effects of fatty acid amide hydrolase catabolic enzyme inhibition in a HIV-1 Tat model of neuroAIDS. Neuropharmacology.

[58] Chicca A., Nicolussi S., Bartholomäus R., Blunder M., Aparisi Rey A., Petrucci V., Reynoso-Moreno I., del Carmen Reynoso-Moreno I., Viveros-Paredes J.M., Dalghi Gens M. (2017). Chemical probes to potently and selectively inhibit endocannabinoid cellular reuptake. Proc. Natl. Acad. Sci. USA.

[59] Metna-Laurent M., Marsicano G. (2015). Rising stars: Modulation of brain functions by astroglial type-1 cannabinoid receptors. Glia.

[60] Ashton J.C. (2012). The use of knockout mice to test the specificity of antibodies for cannabinoid receptors. Hippocampus.

[61] Grimsey N.L., Goodfellow C.E., Scotter E.L., Dowie M.J., Glass M., Graham E.S. (2008). Specific detection of CB1 receptors; cannabinoid CB1 receptor antibodies are not all created equal!. J. Neurosci. Methods.

[62] Chen Y., Liu X., Vickstrom C.R., Liu M.J., Zhao L., Viader A., Cravatt B.F., Liu Q.S. (2016). Neuronal and astrocytic monoacylglycerol lipase limit the spread of endocannabinoid signaling in the cerebellum. eNeuro.

[63] Viader A., Blankman J.L., Zhong P., Liu X., Schlosburg J.E., Joslyn C.M., Liu Q.S., Tomarchio A.J., Lichtman A.H., Selley D.E. (2015). Metabolic interplay between astrocytes and neurons regulates endocannabinoid action. Cell Rep..

[64] Liu X., Chen Y., Vickstrom C.R., Li Y., Viader A., Cravatt B.F., Liu Q. (2016). Coordinated regulation of endocannabinoid-mediated retrograde synaptic suppression in the cerebellum by neuronal and astrocytic monoacylglycerol lipase. Sci. Rep..

[65] Schlosburg J.E., Blankman J.L., Long J.Z., Nomura D.K., Pan B., Kinsey S.G., Nguyen P.T., Ramesh D., Booker L., Burston J.J. (2010). Chronic monoacylglycerol lipase blockade causes functional antagonism of the endocannabinoid system. Nat. Neurosci..

[66] Chao C.-C., Gutiérrez-Vázquez C., Rothhammer V., Mayo L., Wheeler M.A., Tjon E.C., Zandee S.E.J., Blain M., de Lima K.A., Takenaka M.C. (2019). Metabolic control of astrocyte pathogenic activity via cPLA2-MAVS. Cell.

[67] Clarke L.E., Liddelow S.A., Chakraborty C., Münch A.E., Heiman M., Barres B.A. (2018). Normal aging induces A1-like astrocyte reactivity. Proc. Natl. Acad. Sci. USA.

[68] Yun S.P., Kam T.-I., Panicker N., Kim S., Oh Y., Park J.-S., Kwon S.-H., Park Y.J., Karuppagounder S.S., Park H. (2018). Block of A1 astrocyte conversion by microglia is neuroprotective in models of Parkinson’s disease. Nat. Med..

[69] Smith B.C., Sinyuk M., Jenkins J.E., Psenicka M.W., Williams J.L. (2020). The impact of regional astrocyte interferon-γ signaling during chronic autoimmunity: A novel role for the immunoproteasome. J. Neuroinf..

[70] Hindinger C., Bergmann C.C., Hinton D.R., Phares T.W., Parra G.I., Hussain S., Savarin C., Atkinson R.D., Stohlman S.A. (2012). IFN-γ signaling to astrocytes protects from autoimmune mediated neurological disability. PLoS ONE.

[71] Zhang Y., Sloan S.A., Clarke L.E., Caneda C., Plaza C.A., Blumenthal P.D., Vogel H., Steinberg G.K., Edwards M.S., Li G. (2016). Purification and characterization of progenitor and mature human astrocytes reveals transcriptional and functional differences with mouse. Neuron.

[72] Szalai A.J., Hu X., Adams J.E., Barnum S.R. (2007). Complement in experimental autoimmune encephalomyelitis revisited: C3 is required for development of maximal disease. Mol. Immunol..

[73] Scheller A., Kirchhoff F. (2016). Endocannabinoids and heterogeneity of glial cells in brain function. Front. Integr. Neurosci..

[74] Araque A., Castillo P.E., Manzoni O.J., Tonini R. (2017). Synaptic functions of endocannabinoid signaling in health and disease. Neuropharmacology.

