# Supplementary material for: Gene Expression Analysis of Astrocyte and Microglia Endocannabinoid Signaling during Autoimmune Demyelination
Source: Biomolecules. 2020 Aug 24;10(9):1228. doi: 10.3390/biom10091228 (PMC7563448; doi:10.3390/biom10091228)
Supplement: Supplementary file 1 [file biomolecules-10-01228-s001.zip › Supplementary Figure Legends.pdf]

Supplementary Figure 1. FACS sorting and purity of CNS cell populations during EAE. (**A**-**B**) Representative dot-plots depicting FACS strategy for the purification of astrocytes and microglia from EAE mice. Astrocytes were sorted as ACSA-2$^+$ cells following exclusion of contaminating hematopoietic cells (CD45$^+$), microglia/macrophages and hematopoietic cells (CD11b$^+$/CD45$^+$), oligodendrocytes (O1$^+$), oligodendrocyte progenitors (A2B5$^+$) and endothelial cells (CD31$^+$) grouped in a dump channel. *Right*: The purity of sorted astrocyte population by FACS analysis of the astrocytic marker GLAST. (**B**) Microglial cells were sorted as CD11b positive cells with low CD45 expression (CD11b$^+$/CD45^{low}). (**C**) qPCR analysis of astrocyte (*Aldh1l1*, *Aqp4*, *Atp1b2*, *Gfap*, *Slc1a3*) and microglia (*Gpr34*, *Itgam*, *P2ry12*, *Tmem119*) enriched genes in both cell types purified at acute EAE disease (n = 5-6 mice; normalized to astrocytic *Gfap*).

Supplementary Figure 2. Cell-population analysis during EAE. (**A**) Clinical score of the diseased animals used in the study (n = 19 mice; 2 independent EAE experiments). Astrocytes and microglia were sorted from control and immunized mice at the presymptomatic (7-8 dpi), acute (14-17 dpi) and recovery (28-31 dpi). (**B**) Neurological score of the diseased mice used for FACS analysis at each time-point (n = 6 mice). ***p < 0.001; one-way ANOVA followed by Newman-Keuls tests. (**C**) Relative abundance of CD11b$^+$CD45^{high} and CD11b$^-$CD45^{high} peripheral immune cell populations in CNS single-cell suspensions from control and EAE mice at presymptomatic, acute and recovery phases of the disease (n = 6-8 mice per condition). ***p < 0.001 referred to CD11b$^+$ cells, Student´s t-tests.
