# Supplementary figures and images for: Gene Expression Analysis of Astrocyte and Microglia Endocannabinoid Signaling during Autoimmune Demyelination
Source: Biomolecules. 2020 Aug 24;10(9):1228. doi: 10.3390/biom10091228 (PMC7563448; doi:10.3390/biom10091228)

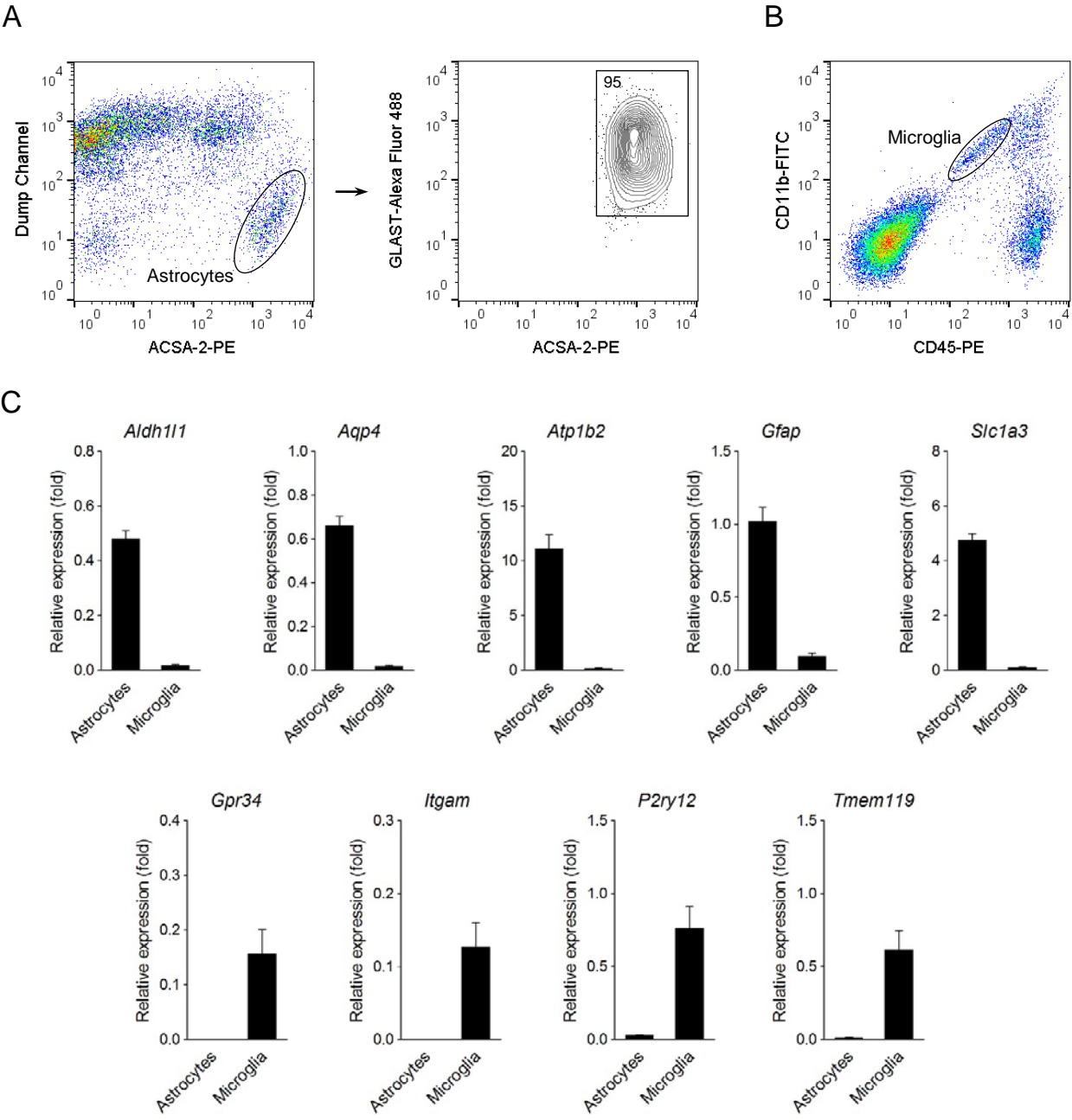

Supplementary Figure 1

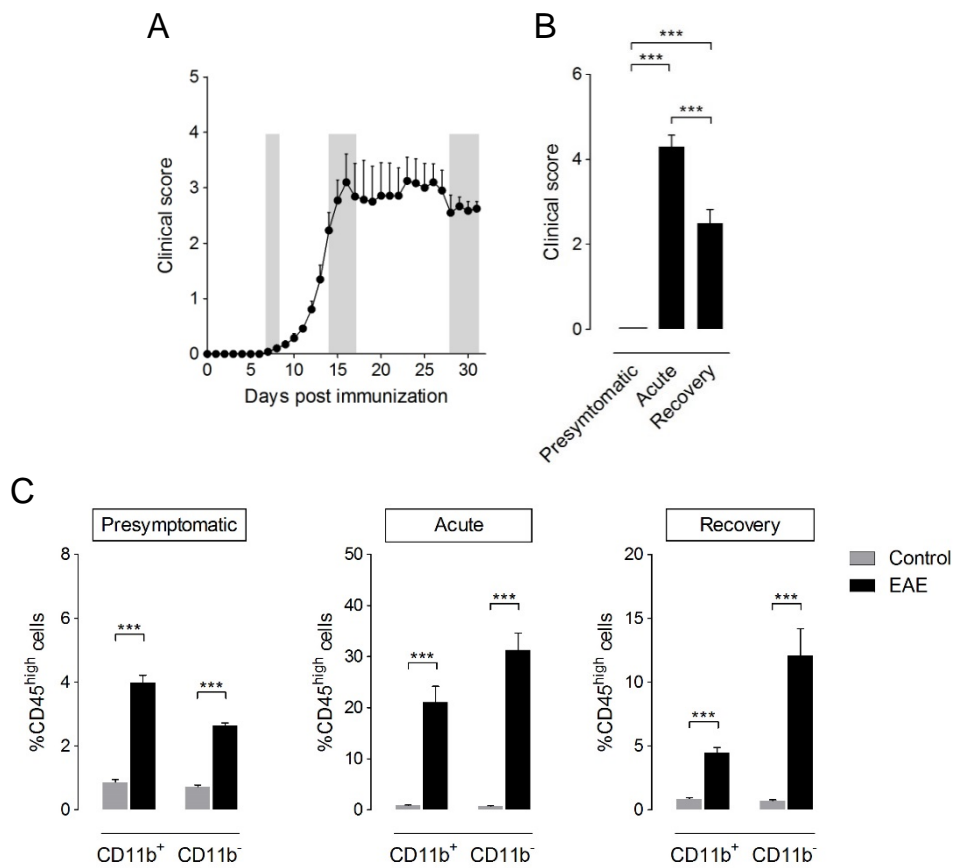

**Supplementary Figure 2**

Supplement: Supplementary file 1 [file biomolecules-10-01228-s001.zip › Moreno-Garcia - Supplementary Figures.pdf]
